# Migration-adjusted prostate cancer incidence in China: a population-based epidemiological analysis

**DOI:** 10.3389/fpubh.2025.1735390

**Published:** 2026-01-21

**Authors:** Yu Zhang, Guijin Li, Shuxiu Hao, Huixin Sun, Linlin Du, Xinyu Liu, Liangfang Xue, Xinshu Wang, Tong Wang, Qi Li

**Affiliations:** 1 Chinese Center for Endemic Disease Control, Harbin Medical University, Harbin, China; 2 NHC Key Laboratory of Etiology and Epidemiology, Harbin Medical University, Harbin, China; 3 Joint Key Laboratory of Endemic Diseases (Harbin Medical University, Guizhou Medical University, Xi’an Jiaotong University), Harbin, China; 4 Institute of Cancer Prevention and Treatment of Heilongjiang Province, Harbin, China; 5 School of Public Health, Qiqihar Medical University, Qiqihar, China; 6 Department of Radiation Oncology, Harbin Medical University Cancer Hospital, Harbin, China; 7 Nanchang University Queen Mary School, Nanchang, China

**Keywords:** cancer surveillance, incidence, INLA-SPDE modeling, migration, prostate cancer, spatial analysis

## Abstract

**Background:**

China’s internal migration poses major challenges to cancer surveillance systems that rely on household-registered populations (HRP). Excluding migrants can lead to biased incidence estimates and misinformed public health planning.

**Methods:**

This study estimated the prostate cancer incidence among the resident population (RP) using a Bayesian integrated nested Laplace approximation with stochastic partial differential equation (INLA-SPDE) model, incorporating inter-provincial migrant weights, and explored spatial clustering.

**Results:**

The findings revealed a substantial interprovincial migrant population of 73,459,708 individuals, based on data from the 2016 China Migrants Dynamic Survey conducted by the Migrant Population Service Center, National Health Commission of China. With Shanghai and Beijing showing relatively high difference proportions of 40.7 and 37.9%, respectively. Nationally, the differences in estimated incidence between RP and HRP were substantial, ranging from 1.1/100,000 in Guizhou (HRP 5.4/100,000, RP 6.5/100,000) to −9.9/100,000 in Shanghai (HRP 27.6/100,000, RP 17.7/100,000). The analysis estimated that the provinces with the largest differences between incident cases among RP and HRP were Guangdong (469 cases, 9.7% relative to HRP cases) and Jiangsu (305 cases, 6.2% relative to HRP cases). Inflow provinces tended to have their cases underestimated and their incidence overestimated, whereas the opposite pattern was observed in outflow provinces. Incidence exhibits significant spatial clustering, with higher incidence in eastern coastal metropolitan areas and lower incidence in central-western regions and northeastern parts.

**Conclusion:**

Migration substantially influences prostate cancer incidence surveillance in China. Incorporating migrant-adjusted estimates provides a more accurate representation of disease burden, supports equitable allocation of healthcare resources, and offers methodological guidance for improving cancer registration systems in regions with high population mobility.

## 1 Introduction

Prostate cancer represents one of the most frequently diagnosed malignancies worldwide. In 2020, approximately 1.41 million new cases were reported, and projections indicate a rise to 2.9 million by 2040 (1–3). Accurate estimation of prostate cancer incidence is essential for understanding disease burden and guiding public health policy. However, in mainland China, such estimation is complicated by large-scale internal migration, which introduces selection bias into cancer surveillance systems based on household registration. Similar issues have been observed in other countries, where migrant populations can distort disease statistics and undermine burden assessment (4).

China’s cancer registries (5–7) traditionally rely on residency-registration data, which fail to capture the rapidly growing migrant population—approximately 376 million individuals, or 26% of the national population in 2020 (8–12). The exclusion of this population limits the accuracy of national cancer estimates and weakens the evidence base for health policy. In contrast, analyses based on the resident population better reflect true environmental exposures and living conditions, as well as potential hormonal and environmental risk factors that may contribute to observed spatial disparities in cancer incidence (13). This offering a more realistic epidemiological foundation for burden assessment and intervention design.

Spatial epidemiology provides powerful tools for identifying geographic disparities and targeting cancer control strategies (14, 15). Nevertheless, to date, no study has examined the spatial distribution of prostate cancer in China using resident-population data. Most existing studies have relied on large-scale registry data that omit migrants, thereby overlooking spatial heterogeneity and mobility-related bias. County-level, small-area analyses can reveal such disparities, refine intervention targets, and provide more precise public health evidence (16).

To overcome these limitations, this study employs a Bayesian integrated nested Laplace approximation with a stochastic partial differential equation (INLA-SPDE) framework to estimate prostate cancer incidence among the resident population in mainland China. Compared with conventional Markov chain Monte Carlo (MCMC) approaches, the INLA-SPDE model offers substantial computational efficiency while preserving the strengths of Bayesian inference (17). It enables high-resolution spatial prediction and uncertainty quantification, making it particularly suitable for nationwide small-area cancer surveillance.

This study estimated prostate cancer incidence and conducted a county-level spatial analysis in China based on migration-adjusted population data. A Bayesian INLA-SPDE framework was applied to estimate prostate cancer incidence using registry-based data, and the model results were combined with inter-provincial migrant weights to obtain migration-adjusted incidence. This approach reduces biases inherent in registry-based surveillance and provides a more accurate understanding of prostate cancer distribution for evidence-based cancer control planning.

## 2 Materials and methods

### 2.1 Study design

We estimated the 2016 prostate cancer incidence among the resident population in mainland China by applying a Bayesian INLA-SPDE model with migration-adjusted weighting. Subsequently, we performed spatial distribution analysis and cluster detection using small-area epidemiology methods.

### 2.2 Data sources

#### 2.2.1 Cancer registries

Prostate cancer incidence data were obtained from 487 registries across 31 provinces in mainland China. These data complied with the quality standards of the International Agency for Research on Cancer (IARC), the International Association of Cancer Registries (IACR), and the criteria in Cancer Incidence in Five Continents, Volume 11 (see Supplementary Table S1 for registry data).

#### 2.2.2 Population

Population data for 2,845 districts/counties in mainland China (2016), including both household-registered and resident populations, were collected from the Population and Employment Statistical Yearbook (10) and the City Statistical Yearbook. Data on interprovincial migrants were obtained from the National Population Census and the China Migrant Dynamic Survey (18) (see Supplementary Table S2 for details).

#### 2.2.3 Covariates

To improve the estimation of prostate cancer incidence, 23 covariates were derived from national census data, statistical yearbooks, and local reports, guided by established epidemiologic evidence. These covariates encompassed population structure, socioeconomic development, education, health resources, housing, air quality, and climate indicators. For example, the proportion of males aged 65 years or older and long-term PM2.5 exposure have been associated with increased prostate cancer risk (19, 20), whereas longer annual sunshine duration may be protective through vitamin D synthesis. Higher incidence rates in economically developed regions may reflect better access to healthcare and screening opportunities (21), while lower educational attainment and smaller per capita housing area, as proxies for socioeconomic status, have been linked to delayed screening and poorer health behaviors (22, 23) (see Supplementary Table S3 for details).

### 2.3 Statistical analysis

#### 2.3.1 Bayesian spatial model

A Bayesian modeling framework was applied to estimate county-level prostate cancer incidence across 2,845 counties, accounting for spatial dependence and overdispersion. The model initially considered 23 standardized covariates, which were screened using the Akaike information criterion (AIC) and further filtered to exclude variables with Pearson correlation coefficients greater than 0.8 or with variance inflation factors (VIF) greater than 10, to avoid multicollinearity (24, 25). The final set of covariates selected was then used in the model. Migration-adjusted estimates of prostate cancer incidence for the overall resident population were subsequently derived by combining the county-level model estimates with interprovincial migrant population data using weighted adjustment. Technical details of the spatial field approximation, including stochastic partial differential equation (SPDE) implementation, mesh construction, and model diagnostics, are provided in the Supplementary Tables S4–S7 and Supplementary Figures S1–S4). Model performance was evaluated using the deviance information criterion (DIC), Watanabe–Akaike information criterion (WAIC), and marginal log-likelihood (MLL).

#### 2.3.2 Sensitivity and validation

We evaluated model robustness through sensitivity analyses across three domains: spatial effects, covariate selection, and parameter settings. We examined spatial dependence by comparing deviance information criterion (DIC) values between models with and without spatial random effects. To quantify the influence of individual covariates, we iteratively removed each predictor from the full model and recorded the resulting DIC changes. We tested parameter sensitivity by varying key settings, including the spatial range (1–10) and partial sill (0.01–5), and validated predictive performance using 5-fold cross-validation (see Supplementary Tables S4–S7 and Supplementary Figures S1–S4).

#### 2.3.3 Migration-adjusted estimation of prostate cancer incidence

We accounted for internal migration by calculating provincial net migrant populations as the difference between the resident and household-registered populations. We then estimated migration-adjusted incidence rates using a weighted approach that reflected the age and origin distribution of migrants within each province. By integrating these adjusted estimates with the household-registered data from the Bayesian model, we obtained the incidence rates for the overall resident population. Finally, we calculated the age-standardized incidence rates (ASIRs) using indirect standardization, with the WHO World Standard Population as the reference population. Age-specific population data were obtained from the Population Pyramid database, and standard incidence rates were obtained from the World Health Organization’s Global Cancer Observatory (see Supplementary Figure S5 for detailed calculation procedures).

#### 2.3.4 Exploratory spatial analysis

We conducted exploratory spatial analyses to visualize geographic patterns and identify clusters of elevated risk. We generated thematic maps to illustrate incidence variation at both provincial and county levels, and we assessed global and local spatial autocorrelation using Moran’s I and Local Indicators of Spatial Association (LISA). These exploratory assessments aimed to support public health interpretation rather than to infer causality.

## 3 Results

### 3.1 Model development

In 2016, prostate cancer incidence data were collected from 487 cancer registries covering both urban and rural areas in mainland China. Bayesian INLA-SPDE models were applied to estimate prostate cancer incidence among the resident population. Considering the study objectives and predictive performance, we selected a final geostatistical model including four covariates: average length of education, per-capita disposable income of urban residents, the proportion of population in the secondary industry, and Urbanization rate of permanent residents. Among them, the estimated coefficient of PerDI on prostate cancer incidence in the house-hold registered population was 0.262 (95% BCI: 0.204–0.319). The standard deviation of the spatial random effect was 0.378 (95% BCI: 0.193–0.908), indicating a significant spatial effect at the overall level. The DIC and WAIC of the final model were 3212.15 and 3186.81, respectively. Model validation showed that the model correctly estimated 96.7% of the locations to fall within the 95% BCI (see Supplementary Figure S4).

### 3.2 Migration-adjusted prostate cancer incident cases

In 2016, an estimated 58,647 new prostate cancer cases were identified in China’s resident population. This represented a net discrepancy of 3,789 cases compared to estimates based on the household-registered population. Discrepancies were strongly associated with internal migration patterns: population inflow provinces like Guangdong showed the largest underestimation (469 cases; 9.7%), whereas outflow provinces like Henan had the largest overestimation (273 cases; 8.6%). At the urban–rural level, the greatest underestimation occurred in urban Guangdong (346 cases; 11.8%), and the greatest overestimation was in rural Henan (228 cases; 10.2%). These results underscore the significant impact of population mobility on the accuracy of regional cancer burden estimates (see Table 1 and Supplementary Figure S6A).

**Table 1:** Provincial differences in estimated prostate cancer incident cases between resident and household-registered populations in mainland China.

Province	Urban				Rural				All			
	HRRP	RP	Diff	Diff (%)	HRRP	RP	Diff	Diff (%)	HRRP	RP	Diff	Diff (%)
Guangdong	2,928	3,274	346	11.8	1,901	2,024	123	6.5	4,829	5,298	469	9.7
Jiangsu	2,669	2,824	155	5.8	2,257	2,407	150	6.6	4,926	5,231	305	6.2
Zhejiang	2,202	2,314	112	5.1	2,869	2,958	89	3.1	5,072	5,272	200	3.9
Shanghai	687	706	19	2.8	1,365	1,503	138	10.1	2,052	2,209	157	7.7
Beijing	1,422	1,488	66	4.6	448	513	65	14.5	1,870	2,001	131	7.0
Fujian	796	851	55	6.9	1,143	1,202	59	5.2	1,940	2,053	113	5.8
Tianjin	414	459	45	10.9	383	426	43	11.2	797	884	87	10.9
Xinjiang	183	210	27	14.8	613	667	54	8.8	796	877	81	10.2
Shanxi	416	443	27	6.5	763	775	12	1.6	1,179	1,219	40	3.4
Hainan	158	170	12	7.6	188	195	7	3.7	347	365	18	5.2
Xizang	17	29	12	70.6	50	53	3	6.0	68	81	13	19.1
Qinghai	58	62	4	6.9	104	109	5	4.8	162	172	10	6.2
Ningxia	129	134	5	3.9	102	107	5	4.9	232	241	9	3.9
Gansu	311	301	−10	−3.2	408	385	−23	−5.6	718	686	−32	−4.5
Yunnan	369	352	−17	−4.6	1,174	1,137	−37	−3.2	1,544	1,489	−55	−3.6
Shaanxi	696	669	−27	−3.9	776	739	−37	−4.8	1,472	1,408	−64	−4.3
Guizhou	353	335	−18	−5.1	884	835	−49	−5.5	1,238	1,171	−67	−5.4
Jiangxi	578	563	−15	−2.6	1,352	1,299	−53	−3.9	1,930	1,862	−68	−3.5
Guangxi	763	741	−22	−2.9	1,059	1,007	−52	−4.9	1,822	1,748	−74	−4.1
Jilin	313	294	−19	−6.1	412	350	−62	−15.0	725	644	−81	−11.2
Inner Mongolia	426	393	−33	−7.7	574	525	−49	−8.5	1,000	917	−83	−8.3
Hebei	931	894	−37	−4.0	1,712	1,662	−50	−2.9	2,643	2,556	−87	−3.3
Liaoning	1,218	1,141	−77	−6.3	648	625	−23	−3.5	1,866	1,765	−101	−5.4
Heilongjiang	547	505	−42	−7.7	597	534	−63	−10.6	1,144	1,039	−105	−9.2
Chongqing	1,318	1,254	−64	−4.9	271	230	−41	−15.1	1,589	1,484	−105	−6.6
Hunan	627	595	−32	−5.1	1,471	1,365	−106	−7.2	2,099	1,959	−140	−6.7
Hubei	948	893	−55	−5.8	1,146	1,047	−99	−8.6	2,094	1,941	−153	−7.3
Anhui	844	803	−41	−4.9	1,642	1,526	−116	−7.1	2,487	2,329	−158	−6.4
Sichuan	1,450	1,373	−77	−5.3	1,789	1,619	−170	−9.5	3,239	2,992	−247	−7.6
Shandong	1,692	1,552	−140	−8.3	2,440	2,317	−123	−5.0	4,132	3,869	−263	−6.4
Henan	928	883	−45	−4.8	2,229	2,001	−228	−10.2	3,157	2,884	−273	−8.6
Emigrating			771				1,381				2,156	
Immigrating			885				753				1,633	
Total	26,394	26,505	1,656	14.4	32,772	32,142	2,134	6.5	59,166	58,647	3,789	6.4

HRRP, household-registered resident population; RP, resident population; Diff, differences in estimated prostate cancer incident cases between the resident and household-registered populations; Diff (%), relative differences in estimated prostate cancer incident cases between resident and household-registered populations.

### 3.3 Migration-adjusted crude incidence of prostate cancer

The estimated crude incidence of prostate cancer was 8.4/100,000 in the resident population. At the provincial level, Shanghai exhibited the most substantial disparity compared to household-registered estimates (−9.9/100,000; −35.9%). This pattern of pronounced disparities was also observed at the urban–rural level, with Beijing showing the largest difference in urban areas (−9.9/100,000; −29.0%) and Shanghai in rural areas (−14.3/100,000; −46.4%). These spatial variations in incidence align with internal migration patterns (see Table 2 and Supplementary Figure S6B).

**Table 2:** Provincial differences in estimated prostate cancer incident between resident and household-registered populations in mainland China.

Province	Urban				Rural				All				Age standardized incidence		
	HRRP	RP	Diff	Diff (%)	HRR	PR	Diff	Diff (%)	HRRP	RP	Diff	Diff (%)	Urban	Rural	All
Guizhou	6.8	6.7	−0.1	−1.5	5.0	6.4	1.4	28.0	5.4	6.5	1.1	20.4	6.8	6.5	6.6
Guangxi	8.1	8.0	−0.1	−1.2	5.4	6.2	0.8	14.8	6.3	6.8	0.5	7.9	8.1	6.3	6.9
Jiangxi	8.0	8.0	0.0	0	7.3	7.9	0.6	8.2	7.5	8.0	0.5	6.7	8.1	8.0	8.1
Anhui	7.8	7.2	−0.6	−7.7	6.6	7.5	0.9	13.6	7.0	7.4	0.4	5.7	7.3	7.6	7.5
Henan	7.7	7.0	−0.7	−9.1	5.0	5.7	0.7	14.0	5.6	6.0	0.4	7.1	7.1	5.8	6.1
Jiangsu	14.8	14.0	−0.8	−5.4	10.7	12.1	1.4	13.1	12.6	13	0.4	3.2	14.1	12.3	13.2
Chongqing	10.7	10.4	−0.3	−2.8	5.6	6.8	1.2	21.4	9.2	9.6	0.4	4.3	10.5	6.9	9.7
Fujian	12.3	10.5	−1.8	−14.6	8.8	10.2	1.4	15.9	10	10.3	0.3	3.0	10.6	10.3	10.4
Qinghai	6.1	5.8	−0.3	−4.9	5.1	5.6	0.5	9.8	5.4	5.7	0.3	5.6	5.9	5.7	5.7
Ningxia	8.4	7.9	−0.5	−6	5.3	6.1	0.8	15.1	6.8	7.0	0.2	2.9	8.0	6.2	7.1
Hebei	9.7	9.4	−0.3	−3.1	6.0	6.1	0.1	1.7	6.9	7.0	0.1	1.4	9.5	6.2	7.1
Jilin	6.8	6.6	−0.2	−2.9	4.8	5.0	0.2	4.2	5.5	5.6	0.1	1.8	6.7	5.0	5.7
Sichuan	9.3	8.5	−0.8	−8.6	5.8	6.2	0.4	6.9	7	7.1	0.1	1.4	8.6	6.3	7.2
Hainan	9.1	7.6	−1.5	−16.5	6.2	7.1	0.9	14.5	7.3	7.3	0.0	0.0	7.7	7.2	7.4
Hubei	10.2	8.3	−1.9	−18.6	4.9	5.4	0.5	10.2	6.4	6.4	0.0	0.0	8.4	5.4	6.5
Hunan	8.6	7.2	−1.4	−16.3	4.9	5.2	0.3	6.1	5.6	5.6	0	0.0	7.2	5.2	5.7
Heilongjiang	8.0	6.7	−1.3	−16.3	5.2	5.6	0.4	7.7	6.2	6.1	−0.1	−1.6	6.7	5.7	6.2
Shandong	8.9	8.3	−0.6	−6.7	7.8	8.0	0.2	2.6	8.2	8.1	−0.1	−1.2	8.4	8.1	8.2
Gansu	7.4	6.5	−0.9	−12.2	4.2	4.2	0.0	0.0	5.2	5.0	−0.2	−3.8	6.6	4.3	5.0
Shanxi	7.8	7.0	−0.8	−10.3	6.2	6.2	0.0	0.0	6.7	6.5	−0.2	−3	7.1	6.3	6.6
Xizang	4.3	3.3	−1.0	−23.3	3.9	4.3	0.4	10.3	4.1	3.9	−0.2	−4.9	3.3	4.4	3.9
Shaanxi	8.4	7.8	−0.6	−7.1	6.5	6.5	0.0	0.0	7.3	7.0	−0.3	−4.1	7.8	6.6	7.1
Yunnan	9.3	7.4	−1.9	−20.4	5.8	5.8	0.0	0.0	6.4	6.1	−0.3	−4.7	7.5	5.9	6.2
Liaoning	11.9	11.3	−0.6	−5.0	5.9	5.8	−0.1	−1.7	8.8	8.4	−0.4	−4.5	11.4	5.9	8.5
Xinjiang	9.7	6.8	−2.9	−29.9	5.9	5.8	−0.1	−1.7	6.5	6.0	−0.5	−7.7	6.9	5.9	6.1
Inner Mongolia	12.1	8.6	−3.5	−28.9	6.4	6.7	0.3	4.7	8.0	7.4	−0.6	−7.5	8.7	6.8	7.5
Guangdong	11.8	8.7	−3.1	−26.3	8.0	10.2	2.2	27.5	9.9	9.2	−0.7	−7.1	8.8	10.3	9.3
Zhejiang	21.7	16	−5.7	−26.3	17.9	18.0	0.1	0.6	19.4	17.0	−2.4	−12.4	16.1	18.2	17.3
Tianjin	15.4	11.4	−4.0	−26.0	14.4	10.6	−3.8	−26.4	14.9	11.0	−3.9	−26.2	11.6	10.7	11.1
Beijing	34.1	24.2	−9.9	−29.0	16.6	10.5	−6.1	−36.7	27.2	18.1	−9.1	−33.5	24.5	10.6	18.3
Shanghai	23.0	20.8	−2.2	−9.6	30.8	16.5	−14.3	−46.4	27.6	17.7	−9.9	−35.9	21.1	16.7	17.9
Total	11.0	9.7	−1.3	−11.8	7.0	7.6	0.6	8.6	8.3	8.4	0.1	1.2	9.8	7.6	8.5

HRRP, household-registered resident population; RP, resident population; ASIR, age-standardized incidence rate; Diff, differences in estimated prostate cancer incidence between the resident and household-registered populations; Diff (%), relative differences in estimated prostate cancer incidence between resident and household-registered population.

### 3.4 Migration-adjusted age-standardized incidence rate

The overall age-standardized incidence rate (ASIR) among the resident population was 8.5/100,000. At the provincial level, Beijing had the highest ASIR (18.3/100,000). From an urban–rural perspective, the highest ASIR was observed in urban Beijing (24.5/100,000), whereas Zhejiang had the highest ASIR in rural areas (18.2/100,000) (see Table 2 and Supplementary Figure S6C).

### 3.5 Spatial distribution of prostate cancer incidence

**Figure 1:**
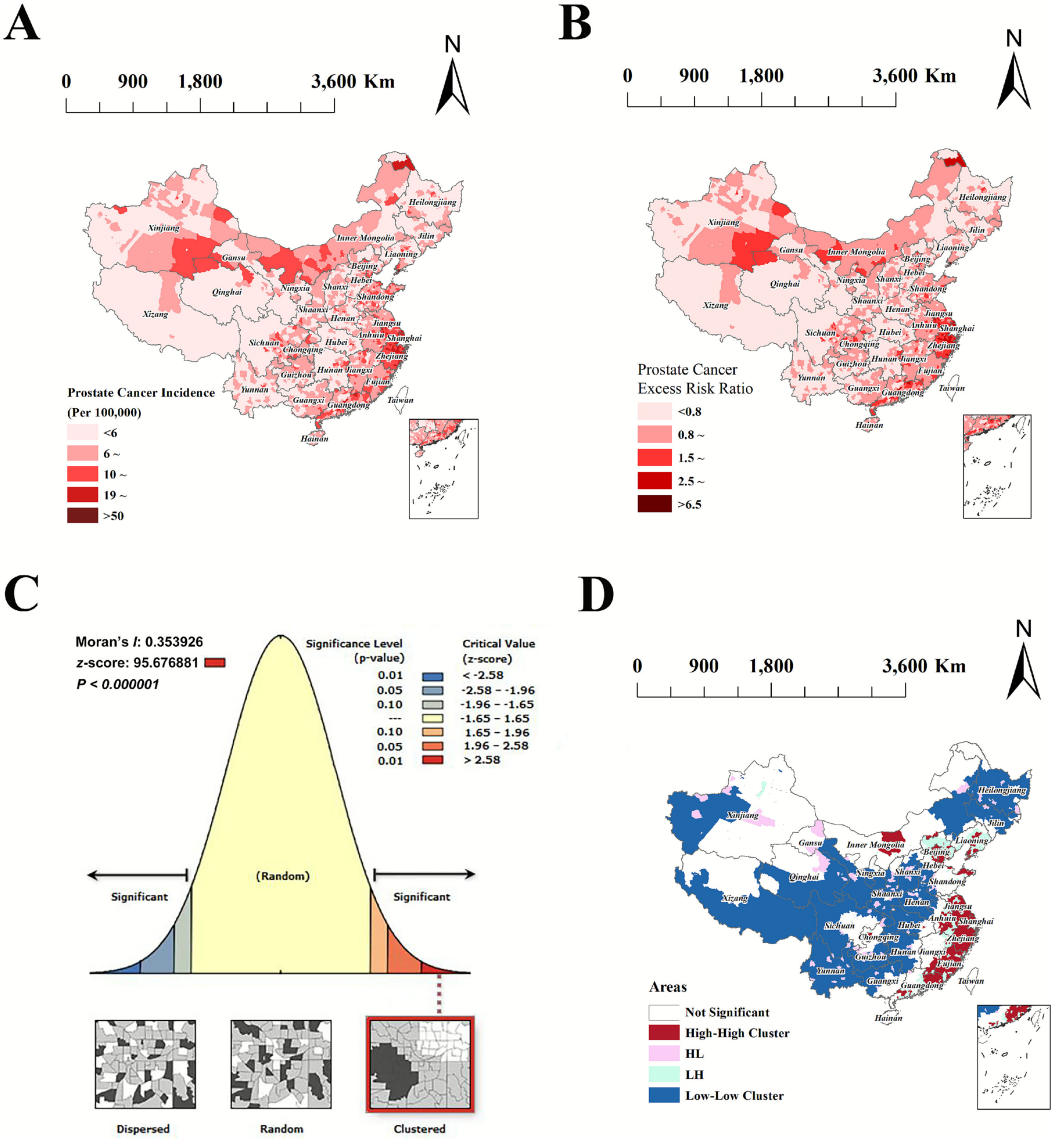
Spatial distribution and spatial autocorrelation of prostate cancer incidence. **(A)** Spatial distribution of prostate cancer incidence. **(B)** Excess risk ratio of prostate cancer incidence. **(C)** Global spatial autocorrelation of prostate cancer incidence. **(D)** Local spatial autocorrelation (LISA) clusters of prostate cancer incidence.

Thematic maps illustrated marked regional variation in prostate cancer incidence across China. Higher rates were concentrated in eastern and northeastern areas, including Guangdong, Jiangsu, Liaoning, Shanghai, and Zhejiang, whereas lower rates were observed in central (Henan, Hubei, Hunan), western (Gansu, Qinghai, Xizang), and southwestern provinces (Sichuan, Yunnan). Overall, these spatial patterns are consistent with population migration flows, with higher incidence in eastern inflow provinces and lower incidence in western outflow provinces (see Figures 1A,B).

### 3.6 Spatial clustering analysis of prostate cancer incidence

The global Moran’s I for prostate cancer incidence was 0.35 (*p* < 0.000001), showing significant spatial autocorrelation. High–high (HH) clusters were concentrated in eastern and northeastern provinces (including Inner Mongolia, Liaoning, Beijing, Shandong, Jiangsu, Zhejiang, Fujian, and Guangdong), while low–low (LL) clusters were primarily located in western and southwestern provinces (including Xizang, Gansu, Yunnan, and Heilongjiang). In HH provinces, the highest proportions of high-incidence counties were observed in Shanghai (100.0%), Zhejiang (100.0%), Beijing (93.8%), and Tianjin (93.8%), with the largest high-incidence populations in Zhejiang (100.0%), Shanghai (100.0%), and Tianjin (97.7%). In LL provinces, Xizang (93.2%), Yunnan (86.0%), and Gansu (83.7%) had the highest proportions of low-incidence counties, while the largest low-incidence populations were found in Xizang (97.6%), Henan (82.0%), and Heilongjiang (80.9%). Other HH or LL provinces showed similar patterns with slightly lower percentages (see Figures 1C,D and Supplementary Table S8).

## 4 Discussion

Prostate cancer incidence estimates in mainland China primarily rely on population-based registry data (26). In this study, we initially estimated prostate cancer incidence using a Bayesian INLA-SPDE model with registered population data, then adjusted the results using inter-provincial migrant population weights to derive incidence estimates for the resident population. This approach accounted for geographic heterogeneity and key risk factors, thereby enhancing estimation accuracy and revealing the influence of population mobility on incidence. Furthermore, provincial-level adjustments and age stratification helped mitigate inherent limitations of conventional modeling approaches. Sensitivity analyses and cross-validation confirmed the robustness of our modeling framework.

Our findings indicate that discrepancies in prostate cancer reporting are strongly associated with population mobility. Cases in provinces experiencing net inflows of migrants, such as Guangdong, were generally underestimated relative to the resident population (RP), whereas cases in net outflow provinces, like Henan, were overestimated. Overall, the total difference in case numbers between the RP and household-registered populations amounted to 3,789 nationally. Similarly, at the urban–rural level, cases in inflow-dominated cities were generally underestimated, while cases in outflow-dominated rural areas were overestimated. This systematic bias highlights the demographic contribution of migrant populations and the need to integrate incidence estimates adjusted for migration into public health planning. Inflow provinces with higher RP case numbers may require strengthened screening programs, enhanced oncology service capacity, and targeted preventive interventions, whereas outflow provinces may need healthcare resource adjustments to better reflect the resident population burden. Incorporating migration-adjusted incidence data provides a scientific basis for precision healthcare strategies, enabling optimal allocation of diagnostic and treatment resources across both sending and receiving regions.

The estimated crude incidence of prostate cancer among the resident population was 8.4/100,000, closely aligning with 8.3/100,000 among the household-registered population. However, provincial-level differences were notable, ranging from 1.1/100,000 in Guizhou to −9.9/100,000 in Shanghai. These spatial discrepancies may reflect interprovincial variation in population mobility and age composition. Urban provinces with substantial net inflow of young male migrants (e.g., Beijing, Shanghai, Tianjin, Guangdong, Zhejiang) exhibited markedly lower incidence among resident populations, as the influx of younger workers reduces the proportion of older adult males and consequently dilutes age-related risk. In contrast, net outflow provinces (e.g., Guizhou, Guangxi, Jiangxi, Henan, Anhui) experienced the opposite effect: outmigration of younger adults leaves behind older residents, elevating prostate cancer incidence among the resident population. Given that over 70% of prostate cancer cases occur in men aged 60 years or older (27), these findings underscore the role of demographic shifts driven by internal migration in shaping regional incidence patterns. Although the national average difference between resident and household-registered populations appears marginal, it should not be regarded as inconsequential. Rather, the observed heterogeneity highlights the need to refine migration-adjusted estimates to more accurately represent the disease burden among the resident population.

Spatial analysis revealed significant clustering of prostate cancer incidence among RP, with a global Moran’s I of 0.35 (*p* < 0.000001), indicating strong positive spatial autocorrelation. High–high (HH) clusters were primarily located in coastal and northeastern regions, including Inner Mongolia, Liaoning, Beijing, Shandong, Jiangsu, Zhejiang, Shanghai, Fujian, and Guangdong, whereas low–low (LL) clusters were concentrated in central and western regions such as Xizang, Qinghai, Gansu, Ningxia, Shaanxi, Henan, Hubei, Hunan, Sichuan, Guizhou, Yunnan, Jilin, and Heilongjiang. HH clusters in inflow provinces may reflect the combined effects of population inflow and expanded resident populations, while LL clusters in outflow regions correspond to reduced population bases. These spatial patterns provide empirical evidence for prioritizing screening, prevention, and healthcare resource allocation.

This study has several public health implications. First, RP-based incidence estimates offer a more accurate assessment of disease burden than HRP-based estimates, particularly in regions with substantial migration. Second, understanding urban–rural disparities and provincial inflow/outflow patterns can guide precision healthcare planning, including optimal distribution of oncology services, screening programs, and targeted interventions. Recent evidence from a large inpatient-based study indicates that healthcare-related travel burden in China varies markedly by region. While average travel time to hospital was moderate (~23 min), a substantial number of patients still required over 1 h of driving to receive care, particularly in remote or resource-poor areas (28). Given our findings of pronounced spatial variation in prostate cancer incidence and the large heterogeneity of resident populations across counties, such disparities in healthcare access may further exacerbate inequalities in early diagnosis, treatment uptake, and disease burden. These observations highlight the importance of region-specific planning for cancer control interventions and equitable allocation of health resources. Third, spatial clustering analysis identifies priority areas for intervention, supporting evidence-based allocation of limited resources. Collectively, these insights highlight the necessity of integrating migration-adjusted incidence into cancer surveillance and policy decision-making, especially in provinces with high population mobility.

Key limitations of this study include the use of 2016 data, which may not fully reflect current incidence patterns, and the limited availability of county-level and age-stratified data, which prevented calculation of directly standardized rates and detailed assessment of additional risk factors such as PSA screening or obesity. Given the continued high population mobility in China, using more recent data (e.g., 2020–2021) might slightly alter provincial-level incidence estimates and could further increase the differences between resident and household-registered populations, particularly in areas with large migrant inflows. Indirect standardization assumes relatively stable age structures across regions. In provinces with substantial young in-migration, this may potentially bias the estimated standardized rates. The reduced proportion of older adults, who are at higher risk, could lead to underestimation of prostate cancer incidence. However, the overall spatial distribution patterns are unlikely to change substantially. Despite these limitations, the Bayesian INLA-SPDE approach provides high-resolution, robust estimates, offering methodological innovations that remain relevant for current and future surveillance efforts. Our study used covariates at the county or group level (e.g., mean sunshine hours, average education), rather than at the individual level. As a result, the findings may be affected by ecological fallacy, where group-level associations do not necessarily reflect individual-level causal relationships. Caution is therefore warranted when interpreting these results, and future studies should consider individual-level data for validation. Additionally, incorporating environmental exposures such as bisphenol A (BPA) as covariates could further enrich the risk modeling by linking population-level incidence data with molecular mechanisms. Future studies should consider including such data to enhance the robustness and explanatory power of spatial prostate cancer analyses.

In conclusion, inter-provincial population mobility substantially affects prostate cancer incidence estimates based on household registration. Incorporating migrant population weights is essential to avoid under- or overestimation. Spatial analysis identified clear geographic clustering, with HH clusters in eastern metropolitan areas and LL clusters in central-western regions. These patterns reflect underlying migration dynamics and offer guidance for targeted prevention and public health interventions. These findings provide critical methodological insights and practical recommendations for cancer surveillance and resource allocation in regions with high population mobility under household registration systems.

## Data Availability

The original contributions presented in the study are included in the article/Supplementary material, further inquiries can be directed to the corresponding authors.
