# Supplementary material for: Migration-adjusted prostate cancer incidence in China: a population-based epidemiological analysis
Source: Front Public Health. 2026 Jan 21;13:1735390. doi: 10.3389/fpubh.2025.1735390 (PMC12868281; doi:10.3389/fpubh.2025.1735390)
Supplement: Supplementary file 1 [file Data_Sheet_1.pdf]

Supplementary Materials

Supplementary Materials 1
Appendix 1. Data Source 2
1.1 Cancer registries 2
1.2 Population 5
1.3 Covariates 7
Appendix 2. Methods 9
2.1 Exploratory analysis 9
2.2 Bayesian INLA-SPDE model 13
2.2.1 Model description 13
2.2.2 Prior distributions 14
2.2.3 Mesh construction 14
2.2.4 Model fitting and selection 16
2.2.5 Model results 18
2.3 Model validation 19
2.4 Sensitivity analysis 20
2.5 Estimated incidence of Prostate cancer in resident population 21
Appendix 3. Result 26

Appendix 1. Data Source

1.1 Cancer registries

The incidence of prostate cancer in mainland China in 2016 was derived from the 2019 China Cancer Registration Annual Report, which had a three-year delay and used the International Classification of Diseases, Tenth Revision (ICD-10) classification to count incidence (code for prostate cancer: C22). The scope of the report covered 31 Chinese provinces and the Xinjiang Production & Construction Corps (excluding Hong Kong, Macao Special Administrative Regions, and Taiwan Province). The data met the requirements set by the International Agency for Research on Cancer (IARC)/International Association of Cancer Registries (IACR) and Cancer Incidence in Five Continents, Volume 11. The criteria for quality control include comparability, completeness and validity of data. The National Cancer Center reviewed cancer registration data submitted by 682 cancer registries, after quality control, included data from 487 cancer registries, which included 669 districts and counties in 31 provinces, representing 23.5% of the 2,845 districts and counties in mainland China. Detailed information on cancer registries in mainland China can be found in Table S1.

Table S1 Prostate Cancer Incidence from Cancer Registries (per 100,000)

Province	Cancer registries PC Incidence/100,000
**Anhui**	Hefei 16.12, Changfeng 8.04, Feidong 10.60, Feixi 9.71, Lujiang 5.49, Chaohu 15.56, Wuhu 12.78, Ma'anshan 16.24, Dangtu 12.68, Tongling 11.12, Yi'an 8.85, Dingyuan 6.17, Tianchang 10.00, Yingzhou 2.84, Yingdong 6.96, Taihe 4.63, Yongqiao 6.79, Lingbi 7.70, Shou 7.08, Jinzhai 5.77, Mengcheng 1.21, Jing 10.28
**Beijing**	Urban 26.20, Suburban 13.36
**Chongqing**	Wanzhou 11.47, Fuling 12.52, Yuzhong 20.53, Jiangbei 18.05, Shapingba 14.43, Jiulongpo 18.01, Nanan 19.97, Beibei 14.36, Dazu 10.83, Yubei 8.83, Banan 12.76, Qianjiang 4.20, Changshou 15.57, Jiangjin 7.84, Hechuan 6.00, Wansheng 13.22, Tongnan 4.95, Tongliang 14.32, Chengkou 3.15, Wulong 5.63, Zhong 8.06, Kaixian 8.32, Yunyang 5.58, Youyang Tujia 5.26
**Fujian**	Fuqing 5.73, Changle 6.87, Xiamen 12.17, Tong'an 6.60, Xiang'an 3.53, Hanjiang 5.43, Yong'an 12.26, Changtai 5.69, Jian'ou 5.94, Xinluo 14.96, Yongding 8.71
**Gansu**	Chengguan 14.89, Qilihe 15.01, Xigu 15.65, Jingtai 5.70, Liangzhou 3.67, Ganzhou 5.35, Jingning 3.62, Dunhuang 2.76, Lintan 1.39
**Guangdong**	Nanxiong 5.19, Shenzhen 7.49, Zhuhai 14.32, Nanhai 12.50, Shunde 11.78, Jiangmen 11.34, Duanzhou 18.82, Sihui 10.53, Meixian 13.18, Dongguan 16.06, Zhongshan 13.98, Jiexi 8.53, Luoding 3.33, Urban Guangzhou 23.95, Suburban Guangzhou 13.33
**Guangxi**	Xingning 16.73, Qingxiu 10.81, Jiangnan 4.35, Xixiangtang 15.78, Binyang 3.74, Liuzhou 13.44, Guilin 13.34, Wuzhou 11.03, Cangwu 6.44, Beihai 6.20, Hepu 3.53, Gangnan 7.70, Qintang 12.40, Pingnan 5.82, Youjiang 7.27, Lingyun 4.35, Heshan 4.93, Fusui 1.64

**Guizhou**	Huaxi 7.39, Kaiyang 8.57, Qingzhen 6.94, Zhongshan 10.57, Liuzhi 4.16, Chishui 4.89, Xixiu 7.00, Zhenning Buyi 3.61, Bijiang, Tongren 4.95
**Hainan**	Qionghai 9.49, Ding'an 5.70, Changjiang Li Autonomous 8.02, Lingshui Li Autonomous 7.71
**Hebei**	Zanhuang 2.14, Xinji 8.17, Qianxi 4.15, Qian'an 1.78, Qinhuangdao 10.57, Daming 1.70, She 0.91, Ci 0.62, Wu'an 0.89, Xingtai 6.46, Xingtai (County) 2.76, Lincheng 2.69, Neiqiu 2.88, Ren 3.49, Baoding 12.33, Wangdu 2.20, Anguo 0.52, Xuanhua 6.45, Zhangbei 6.43, Shuangqiao 3.84, Fengning Manchu 5.66, Cangzhou 6.25, Haixing 0.89, Yanshan 1.35, Jizhou 4.89, Urban Shijiazhuang 9.86, Suburban Shijiazhuang 4.87
**Heilongjiang**	Daoli 10.36, Nangang 10.34, Xiangfang 9.57, Dong'an 8.04, Yangming 5.26, Aimin 8.38, Xi'an 9.08, Hailin 5.49
**Henan**	Gongyi 8.94, Xiangfu 4.35, Luoyang 11.64, Mengjin 7.32, Xin'an 9.38, Luanchuan 3.70, Song 4.11, Ruyang 2.96, Luoning 0.38, Yichuan 3.00, Yanshi 7.83, Lushan 2.46, Linzhou 2.81, Hebi 2.41, Huixian 4.21, Hualong 14.75, Puyang 4.08, Yuzhou 2.08, Yuanhui 5.95, Yancheng 5.04, Zhaoling 3.26, Hubin 10.22, Wolong 4.46, Fangcheng 2.97, Neixiang 3.50, Sui 3.13, Yucheng 2.76, Shihe 2.99, Luoshan 3.78, Shenqiu 2.49, Dancheng 1.83, Xiping 5.27, Jiyuan 3.54
**Hubei**	Wuhan 19.49, Daye 6.36, Yun 2.71, Yichang 12.13, Miao Autonomous 3.87, Xiangyang 10.83, Yicheng 6.09, Jingshan 5.13, Zhongxiang 5.49, Yunmeng 6.06, Gong'an 8.01, Honghu 5.21, Macheng 5.32, Jiayu 2.57, Enshi 7.22, Tianmen 7.07
**Hunan**	Furong 10.07, Tianxin 14.52, Yuelu 8.38, Kaifu 10.49, Yuhua 11.52, Wangcheng 4.79, Lusong 8.05, Shifeng 16.50, You 4.33, Yuhu 8.81, Hengdong 2.03, Shaodong 5.64, Yueyanglou 6.81, Wuling 6.17, Cili 2.49, Ziyang 6.48, Taojiang 3.46, Linwu 1.94, Zixing 3.08, Xintian 0.86, Mayang Miao Autonomous 2.37, Lianyuan 4.49
**Inner Mongolia**	Tumote Right Banner 5.21, Chifeng 7.47, Aohan 4.72, Horqin Left Middle Banner 2.64, Kailu 3.00, Kulun 3.42, Naiman 0.50, Jarud 0.66, Hailaer 10.83, Arong 2.84, Ewenki Autonomous Banner 4.35, Manzhouli 2.41, Yakeshi 6.68, Genhe 3.64, Linhe 4.97, Xilinhot 7.00
**Jiangsu**	Wuxi 28.32, Jiangyin 18.93, Yixing 13.13, Xuzhou 15.14, Changzhou 27.31, Liyang 15.16, Jintan 23.10, Suzhou 29.48, Changshu 23.54, Zhangjiagang 23.17, Kunshan 30.35, Taicang 37.37, Nantong 26.48, Haian 22.54, Rudong 18.08, Qidong 25.71, Rugao 15.95, Haimen 15.51, Lianyungang 9.56, Ganyu 3.28, Donghai 3.89, Guanyun 5.24, Guannan 4.35, Huai'an 5.84, Huaiyin 4.75, Qingjiangpu 7.49, Lianshui 3.68, Hongze 1.55, Xuyi 8.51, Jinhu 5.62, Tinghu 10.01, Yandu 8.05, Binhai 5.12, Funing 3.34, Sheyang 7.90, Jianhu 7.05, Dongtai 8.84, Dafeng 15.92, Baoying 7.57, Danyang 14.97, Yangzhong 5.07, Taixing 5.84
**Jiangxi**	Wanli 5.71, Xinjian 7.11, Changjiang 3.70, Luxi 5.15, Xunyang 9.74, Wuning 4.46, Yushui 6.53, Zhanggong 13.03, Gan 5.87, Dayu 4.62, Shangyou 4.40, Chongyi 3.05, Longnan 5.19, Anfu 5.98, Wanzai 4.44, Shanggao 1.17, Jing’an 7.60, Dongxiang 0.00, Xinzhou 14.88, Shangrao 2.20, Yanshan 4.00, Hengfeng 8.23, Yugang 3.98, Wannian 5.25, Wuyuan 6.33
**Jilin**	Dehui 3.16, Jilin 9.93, Yongji 2.49, Jiaohe 2.21, Tonghua 1.62, Meihekou 4.71, Fusong 6.62, Ningjiang 3.52, Qian Gorlos Mongolian Autonomous 1.02, Qian'an 2.88, Yanji 5.40, Tumen 5.05, Dunhua 3.78, Helong 6.52, Wangqing 6.87
**Liaoning**	Shenyang 12.98, Kangping 5.13, Faku 3.06, Dalian 21.54, Zhuanghe 9.96, Anshan 12.59, Benxi 7.94, Dandong 9.96, Donggang 7.32, Yingkou 7.30, Fuxin 9.15, Zhangwu 2.44,

	Liaoyang 2.84, Dawa 6.31, Jianping 2.33
**Ningxia**	Yinchuan 9.04, Helan 6.01, Dawukou 10.71, Huinong 15.85, Pingluo 4.44, Qingtongxia 9.35, Zhongwei 4.35, Zhongning 7.79
**Qinghai**	Xining 11.11, Huangzhong 2.03, Ledu 2.70, Hainan Tibetan Autonomous Prefecture 5.53
**Shaanxi**	Beilin 15.98, Lianhu 17.06, Weiyang 4.09, Yanta 12.14, Gaoling 6.06, Hu 4.10, Wangyi 6.76, Jintai 7.40, Qianyang 4.66, Linyou 0.00, Linwei 6.16, Huaxian 1.81, Hanbin 2.35, Ningshan 7.81, Ziyang 3.30, Xunyang 4.75, Shangzhou 3.39
**Shandong**	Jinan 15.38, Zhangqiu 7.65, Qingdao 12.33, Huangdao 7.27, Linzi 15.01, Yiyuan 12.85, Tengzhou 6.51, Yantai 12.37, Zhaoyuan 10.33, Linqu 6.20, Gaomi 5.60, Wenshang 4.06, Liangshan 3.97, Qufu 6.73, Zoucheng 5.15, Ningyang 6.12, Feicheng 7.60, Rushan 10.69, Yinan 7.33, Junan 3.89, Bincheng 9.07, Shan 4.14, Juye 3.15
**Shanghai**	Shanghai 51.67
**Shanxi**	Xinghualing 9.60, Yangquan 5.52, Pingding 1.87, Pingshun 0.00, Yangcheng 1.04, Xiyang 2.43, Shouyang 4.51
**Sichuan**	Qingyang 23.02, Longquanyi 13.04, Pengzhou 11.87, Ziliujing 17.58, Gongjing 15.32, Yantan 6.51, Renhe 10.26, Longmatan 4.99, Luxian 4.33, Hejiang 5.76, Jingyang 7.99, Luojiang 7.07, Guanghan 11.81, Shifang 11.08, Mianzhu 14.72, Youxian 9.03, An 4.40, Santai 3.87, Yanting 1.60, Beichuan Qiang Autonomous 2.46, Chaotian 3.71, Wangcang 10.62, Qingchuan 1.72, Jiange 2.94, Shehong 1.80, Dongxing 6.37, Zizhong 5.22, Shizhong 13.63, Hawan 8.42, Jiajiang 5.78, Gaoping 8.39, Langzhong 7.29, Dongpo 10.23, Renshou 7.79, Qingshen 4.92, Cuiping 10.80, Changning 3.65, Guang'an 3.42, Dazhu 4.58, Yucheng 8.99, Mingshan 14.68, Yingjing 5.34, Hanyuan 15.17, Shimian 8.04, Tianquan 6.31, Lushan 8.07, Baoxing 3.34, Yanjiang 10.16
**Tianjin**	Urban Tianjin 17.25, Suburban Tianjin 8.05
**Xinjiang**	Tianshan 15.87, Midong 8.72, Karamay 12.56, Seventh Division 4.52, Eighth Agricultural Division 19.43, Lhasa 1.91
**Yunnan**	Panlong 17.48, Guandu 21.19, Xishan 16.76, Dongchuan 8.28, Zhanyi 0.00, Hongta 10.95, Jiangchuan 8.49, Chengjiang 8.37, Yimen 8.30, Eshan Yi Autonomous 7.69, Xinping Yi and Dai Autonomous 3.52, Longyang 9.81, Shidian 6.79, Tengchong 2.61, Yulong Naxi Autonomous 6.24, Chuxiong 5.25, Gejiu 9.24, Kaiyuan 11.23, Mengzi 8.93, Pingbian Miao Autonomous 3.59, Shiping 3.74, Luxi 3.64, Jinghong 9.95, Lanping Bai and Pumi Autonomous 1.72
**Zhejiang**	Hangzhou 30.05, Yinzhou 35.48, Cixi 20.97, Lucheng 23.02, Jiaxing 37.83, Jiashan 36.09, Haining 28.76, Changxing 22.79, Shangyu 20.21, Yongkang 20.85, Kaihua 10.77, Daishan 21.79, Xianju 16.96, Longquan 13.48
**Xizang**	Lasa1.91

1.2 Population

According to the Household Registration Management Regulations of the People's Republic of China, household-registered residents refer to citizens officially registered as residents by local public security household registration authorities. This population category includes both individuals physically residing in the registration locality and those temporarily relocating for employment, education, or other purposes, while excluding individuals whose household registration has lost due to outmigration. Regardless of whether or not they reside there, as long as they have registered their residence in a given area, they are considered part of the registered population of that area. China's resident population is defined as individuals who have actually resided in a given area for a specific period of time, usually more than six months. This group includes individuals who live locally but are registered or pending registration in the same area, those who live locally but are temporarily absent from the area of their household registration for more than six months, and individuals with local household registration who are temporarily absent from the area for less than six months or who are working or studying abroad. The net migrating population is defined as the difference between the resident population and the household-registered resident population. The net migrating population can be positive or negative, indicating whether the region is a immigrating region or a emigrating region. Population information for 31 provinces in mainland China in 2016 were shown in Table S2.

Table S2 Population Distribution by Province in Mainland China

Province	Male			
	HRR	PR	Difference	Relative difference (%)
Guangdong	48,599,089	57,460,741	8,861,652	15.4
Shanghai	7,424,017	12,515,117	5,091,100	40.7
Zhejiang	26,140,945	30,927,356	4,786,411	15.5
Beijing	6,866,297	11,053,440	4,187,143	37.9
Tianjin	5,344,343	8,031,849	2,687,506	33.5
Xinjiang	12,331,162	14,520,938	2,189,776	15.1
Shanxi	17,684,891	18,718,108	1,033,217	5.5
Jiangsu	39,170,760	40,085,405	914,645	2.3
Fujian	19,407,822	19,959,749	551,927	2.8
Xizang	1,672,969	2,090,793	417,824	20.0
Hainan	4,784,954	4,990,781	205,827	4.1
Qinghai	2,992,105	3,026,574	34,469	1.1
Ningxia	3,434,742	3,446,368	11,626	0.3

**Yunnan**	24,290,871	24,250,536	-40,335	-0.2
**Inner Mongolia**	12,507,337	12,400,265	-107,072	-0.9
**Gansu**	13,897,282	13,763,306	-133,976	-1.0
**Shaanxi**	21,139,618	20,904,157	-235,461	-1.1
**Liaoning**	20,221,503	19,976,953	-244,550	-1.2
**Heilongjiang**	18,367,446	17,031,875	-1,335,571	-7.3
**Hebei**	38,378,068	36,684,898	-1,693,170	-4.4
**Chongqing**	17,185,140	15,419,779	-1,765,361	-10.3
**Jilin**	13,247,298	11,478,408	-1,768,890	-13.4
**Hubei**	32,508,177	30,237,776	-2,270,401	-7.0
**Jiangxi**	25,669,755	23,365,587	-2,304,168	-9.0
**Shandong**	50,220,107	47,660,182	-2,559,925	-5.1
**Hunan**	37,533,901	34,795,730	-2,738,171	-7.3
**Guangxi**	29,133,594	25,581,883	-3,551,711	-12.2
**Sichuan**	46,280,066	42,264,720	-4,015,346	-8.7
**Anhui**	35,695,228	31,590,739	-4,104,489	-11.5
**Guizhou**	22,764,940	18,063,540	-4,701,400	-20.7
**Henan**	56,746,556	47,829,968	-8,916,588	-15.7
**Emigration Subtotal**			-42,486,585	
**Immigration Subtotal**			30,973,123	
**Total**	711,640,983	700,127,521	73,459,708	10.3

1.3 Covariates

We extracted data on 23 covariates from national population censuses and local yearbooks to estimate prostate cancer incidence among household-registered populations. These covariates covered a wide range of fields, including demography (proportion of ethnic minority population, urban and rural population, population aged 15 years and over, urbanization rate of resident population, Urban permanent population), economy (GDP, per capita GDP, per capita disposable income of urban residents, proportions of population in primary, secondary, and tertiary industries), education (average years of education, illiteracy rate), marriage (populations of unmarried, married, divorced,), housing (mean number of rooms per household, Per capita housing construction area), air quality (PM2.5), and meteorology (mean sunshine hours, mean temperature, and mean precipitation). The information of covariates is shown in

Table S3 Covariates

Category	Covariates	Source	Temporal resolution	Collection Method
Demography	Proportion of ethnic minority population	Population Census	Annual	Interpolation
	Urban population	Population Census	Annual	Interpolation
	Rural population	Population Census	Annual	Interpolation
	Population aged 15 years and over	Population Census	Annual	Interpolation
	Urbanization rate of resident population	Urban Statistical Yearbook; Statistical Bulletin	Annual	Direct access
	Urban permanent population	Urban Statistical Yearbook; Statistical Bulletin	Annual	Direct access
Economy	Gross Domestic Product (GDP)	Urban Statistical Yearbook; Statistical Bulletin	Annual	Direct access
	Per capita GDP	Urban Statistical Yearbook; Statistical Bulletin	Annual	Direct access
	Per capita disposable income of urban residents	Urban Statistical Yearbook; Statistical Bulletin	Annual	Direct access
	Proportions of population in primary industry	Population Census	Annual	Extrapolation
	Proportions of population in secondary industry	Population Census	Annual	Extrapolation
	Proportions of population in tertiary industry	Population Census	Annual	Extrapolation

**Education**	Average years of education	Population Census	Annual	Interpolation
	Illiteracy rate	Population Census	Annual	Interpolation
**Marriage**	Unmarried population	Population Census	Annual	Interpolation
	Married population	Population Census	Annual	Interpolation
	Divorced population	Population Census	Annual	Interpolation
**Housing**	Mean number of rooms per household	Population Census	Annual	Direct access
	Per capita housing construction area (m2/person)	Population Census	Annual	Direct access
**Air quality**	PM2.5	Ministry of Ecology and Environment of the People's Republic of China	Static	Direct access
**Meteorology**	Mean sunshine hours	Urban Statistical Yearbook	Annual	Direct access
	Mean temperature	Urban Statistical Yearbook	Annual	Direct access
	Mean precipitation	Urban Statistical Yearbook	Annual	Direct access

Appendix 2. Methods

2.1 Exploratory analysis

As an initial stage of geostatistical analysis, exploratory analysis involves visualization of the distribution of sampled locations. Following data cleaning, if specific geographic information on certain registries were lacking at the national level, these locations are merged, resulting in a total of 483 cancer registries. A covariate selection process was carried out to determine the most appropriate set of covariates for modelling. This process entailed separately assessing the relationship between the covariates and the incidence of prostate cancer in the household-registered resident population. To eliminate the effect of magnitude among different covariates and ensure comparability, they were converted to standardized continuous variables with a mean of zero and a standard deviation of one. First, we fitted univariate models based on the predictive strength of the covariates, and statistically significant covariates were ranked from low to high according to the Akaike information criterion (AIC). The significant covariates associated with the incidence of prostate cancer were Mean sunshine hours, Mean temperature, Mean precipitation, Average years of education, GDP, Per capita GDP, Per capita disposable income of urban residents, Proportion of ethnic minority population, Urban population, Rural population, Illiteracy rate, Population aged 15 years and over, Unmarried population, Married population, Divorced population, Proportions of population in primary industry, Proportions of population in secondary industry, Proportions of population in tertiary industry, Urban permanent population, Urbanization rate of resident population. Information on significant covariates associated with the estimation model for prostate cancer incidence was shown in Table S4.

We checked for multicollinearity among covariates by calculating Pearson correlations and variance inflation factors (VIF) for each pair of covariates. In cases where covariates exhibited high correlation (correlation coefficient > 0.8 and VIF > 10), we made selections based on meaningfulness or better data quality. The final set of covariates in the estimation model for the male incidence of prostate cancer included Proportion of ethnic minority population, Average years of education, Per capita GDP, Divorced population, Mean temperature, Mean precipitation, Urbanization rate of resident population, Mean sunshine

hours, Illiteracy rate, Per capita disposable income of urban residents, Rural population, Proportions of population in primary industry, Proportions of population in secondary industry and above. Detailed information on the covariates screened by the estimation model for prostate cancer incidence is shown in Table S5.

Table S4 Univariate Correlation Analysis Results

Variables	Estimate	Std. Error	z value	*P*	AIC
Mean sunshine hours	-0.148	0.035	-4.265	<0.001	4095.10
Mean temperature	0.201	0.037	5.448	<0.001	4084.30
Mean precipitation	0.232	0.034	6.907	<0.001	4065.90
Average years of education	0.279	0.035	8.030	<0.001	4049.10
Gross Domestic Product (GDP)	0.132	0.014	9.464	<0.001	4034.50
Per capita GDP	0.362	0.026	13.930	<0.001	3975.20
Per capita disposable income of urban residents	0.389	0.026	14.940	<0.001	3906.60
Proportion of ethnic minority population	-0.197	0.053	-3.691	<0.001	4101.30
Urban population	0.153	0.016	9.558	<0.001	4031.80
Rural population	0.179	0.031	5.861	<0.001	4076.70
Illiteracy rate	-0.589	0.100	-5.902	<0.001	4080.90
Population aged 15 years and over	0.162	0.018	8.782	<0.001	4035.40
Unmarried population	0.141	0.017	8.200	<0.001	4048.80
Married population	0.165	0.019	8.840	<0.001	4033.50
Divorced population	0.170	0.017	10.050	<0.001	4032.00
Proportions of population in primary industry	0.315	0.028	11.160	<0.001	3989.50
Proportions of population in secondary industry	-0.435	0.027	-15.920	<0.001	3898.00
Proportions of population in tertiary industry	0.313	0.029	10.630	<0.001	4020.00
Urban permanent population	0.139	0.017	8.703	<0.001	4044.30
Urbanization rate of resident population	0.324	0.032	10.070	<0.001	4018.30

Table S5 VIF analysis

Variables	VIF
Proportion of ethnic minority population	4.185
Average years of education	5.612
Per capita GDP	4.859
Divorced population	4.999
Mean temperature	6.776
Mean precipitation	6.314
Urbanization rate of resident population	4.717
Mean sunshine hours	5.622
Illiteracy rate	4.535
Per capita disposable income of urban residents	4.940
Rural population	4.681
Proportions of population in primary industry	7.872
Proportions of population in secondary industry	6.744

2.2 Bayesian INLA-SPDE model

2.2.1 Model description

We established Bayesian INLA-SPDE models to predict the prostate cancer incidence of the household-registered resident population of 2,845 districts and counties using the prostate cancer incidence of the 487 cancer registries and quantified the impact of each covariate on the prostate cancer incidence. The INLA-SPDE models were implemented using the R-INLA program package for R 4.3.2. The mathematical forms are as following:

$$Y_i \sim Poisson(m_i \cdot P_i)$$

$$Log(Y_i) = offset\big(log(P_i)\big) + \beta_0 + \beta_1 X_1 + \ldots + \beta_n X_n + f\big(X_{ID,}iid\big) + f(w, SPDE)$$

where Y_i and P_i represent the number of prostate cancer cases/number of deaths and population in the cancer registries, respectively; mi represents the incidence of prostate cancer in the cancer registries; *offset*(*log*(P_i)) represents the offset term; β_0 represents the intercept term, which represents the log incidence at the baseline level; $\beta_1, \cdots, \beta_n$ are the regression coefficients of the fixed effects; *f* (*XID, iid*) represents each cancer registry obeying an independent homogeneous distribution to address overdispersion; w is a spatial random effect, constructed via a linear fractional-order stochastic partial differential equation (SPDE), used to capture spatial dependence.

Gaussian fields with a Matérn covariance function model are smooth solutions of the SPDE:

$$(\kappa^2 - \Delta)^{\frac{\alpha}{2}}\big(\tau \times \xi(s)\big) = W(w)$$

where Δ is the Laplace operator; κ is the scale parameter, associated with the empirical variant r, $r = \sqrt{8v}/\kappa$; α is associated with the smoothing parameter v of the Matérn covariance function, $\alpha = v + d/2$ (d is the spatial dimensionality); τ is the parameter controlling the variance of the Gaussian random field; $\xi(s)$ is a smooth Matérn Gaussian random field; and $W(w)$ is spatial white noise.

The finite element method is used to solve stochastic partial differential equations, triangulating the study area and approximating Matérn Gaussian random fields with the basis functions of the constrained subdivided Delaunay triangular mesh and the weights of the Gaussian distribution:

$$\xi(s) = \sum_{m=1}^{M} \varphi_m(s)\widetilde{\xi_m}$$

where m is the number of MESH vertices of the Delaunay triangulation network; $\varphi_m(s)$ is a set of segmented basis functions; ξ_m denotes the weights with mean 0 having a Gaussian distribution obeying the probability distribution of *Normal* $(0,Q^{-1})$; and Q is the precision matrix, controlled by the scale parameter κ and the variance parameter τ. When α is equal to 2, the precision matrix formula is:

$$Q = \tau^2(k^4C + 2k^2G + GC^{-1}G)$$

where C is a diagonal matrix with the number of rows and columns equal to the number of nodes of the triangular network MESH. G is a sparse matrix with the number of rows and columns also equal to the number of nodes.

2.2.2 Prior distributions

In the INLA method, the prior for the latent Gaussian field of the hidden Gaussian model is specified as a Gaussian Markov Random Field (GMRF), and the remaining task is to choose the prior distribution for the hyperparameters. The internal representation of SPDE parameters is expressed in logarithmic form as $theta_1=\log(\tau)$ and $theta_2=\log(\kappa)$, with the assumption that $theta_1$ and $theta_2$ are independent. The range parameter of the estimation model for incidence of Prostate cancer was set to mean 0.1 and standard deviation 0.1, while the sigma parameter was set to mean 2 and standard deviation 0.1. The intercept and covariate coefficients for fixed effects are both specified with a prior distribution having a mean of 0 and precision of 0.001.

2.2.3 Mesh construction

Based on the semi variogram of the incidence for registered population across 483 cancer registries, an exponential model was employed as the theoretical model for the semi variogram. The fitting results indicated an effective range r of 7 km for male incidence of Prostate cancer, as shown in Figure S1. This information was used as prior knowledge to construct a Delaunay triangulation mesh. For the estimation model for incidence of Prostate cancer, the maximum edge length of inner grid triangles was set to 1.0 degree, and the maximum edge length of the buffer zone was set to 3.0 degree. as illustrated in Figure S2.

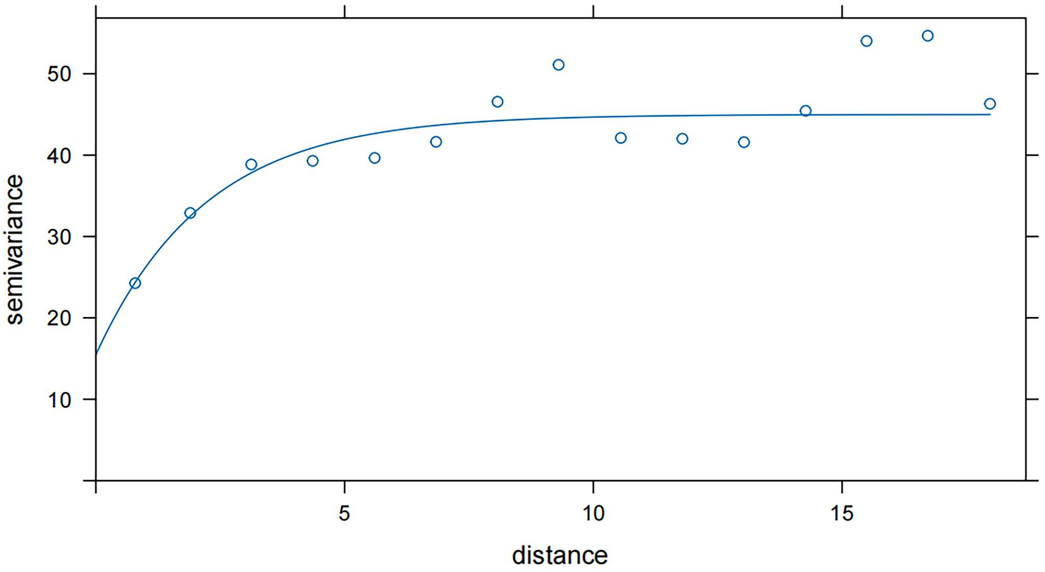

Fig. S1 Semi-variogram plot showing residual autocorrelation of Prostate cancer incidence based on Bayesian model

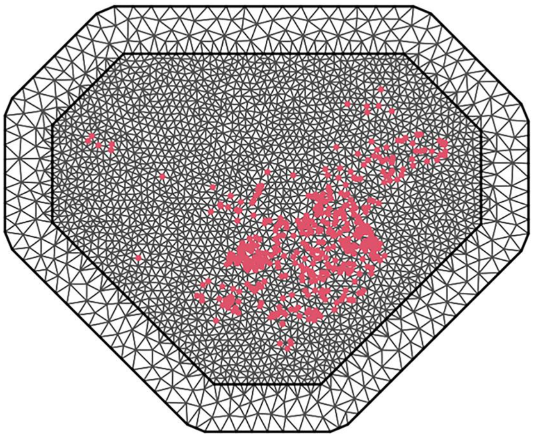

Fig. S2 Finite elements mesh for estimation model of Prostate incidence

2.2.4 Model fitting and selection

The model conforms to the methodology of Integrated Nested Laplace Approximations (INLA). To determine the importance of considering spatial modeling, a single modeling framework excluding the spatial component was initially used to fit the indicator. In the estimation model of prostate cancer incidence, DIC = 3212.15 for the model with spatial random effects and DIC = 3273.62 for the model without spatial random effects, indicating spatial heterogeneity.

Additionally, to assess whether covariates improved the prediction of prostate cancer incidence in the Bayesian INLA-SPDE model, we compared the deviance information criterion (DIC), widely applicable information criterion (WAIC), and marginal log likelihood (MLL) of the Bayesian INLA-SPDE model with and without covariates. Considering the research objectives and predictive capabilities, the estimation model for prostate cancer incidence ultimately selected the following variables: length of education, per-capita disposable income of urban residents, the proportion of population in the secondary industry, and urbanization rate of permanent residents. The results of variable selection for all estimation models were detailed in Table S6.

Table S6 Model Fit Indices (DIC, WAIC, MLL) of INLA-SPDE Bayesian Models with Different Covariates for Prostate Cancer Incidence

Model	Covariates															DIC	WAIC	MLL
	a	b	c	d	e	f	g	h	i	j	k	l	m	n	o			
Model 1	√	√	√	√	√	√	√	√	√	√	√	√	√	√	√	3252.04	3220.09	-1941.62
Model 2	√	√	√	√	√	√		√	√	√	√	√	√	√	√	3252.00	3220.62	-1936.06
Model 3	√	√	√	√	√	√			√	√	√	√	√	√	√	3252.27	3221.11	-1930.45
Model 4	√	√	√	√	√	√				√	√	√	√	√	√	3252.04	3217.90	-1923.62
Model 5	√	√	√	√	√	√					√	√	√	√	√	3251.30	3218.48	-1919.33
Model 6															√	3816.28	4568.90	-2216.29
Model 7	√	√	√	√	√	√	√	√	√	√	√	√	√	√		3273.62	3227.02	-1995.89
Model 8	√	√	√	√	√	√						√	√	√	√	3251.40	3219.40	-1911.38

Model 9	√	√	√	√	√	√						√	√	√		3252.03	3220.94	-1905.75
Model 10	√	√	√	√	√	√							√	√		3203.13	3240.51	-1958.87
Model 11		√	√	√	√	√							√	√		3237.81	3202.36	-1953.76
Model 12	√		√	√	√	√							√	√		3256.91	3227.44	-1902.57
Model 13	√	√	√	√									√	√		3212.15	3186.81	-1971.67
Model 14	√			√		√										3255.11	3209.97	-2023.88
Model 15		√			√	√										3275.14	3229.78	-1941.59
Model 16		√			√											3218.80	3188.57	-1947.10

Notes: a Average years of education; b Per capita disposable income of urban residents; c Proportions of population in secondary industry; d Urbanization rate of permanent residents; e Proportions of population in primary industry; f Rural population; g Proportion of ethnic minority population; h Mean sunshine hours; i Per capita GDP; j Illiteracy rate; k Divorced population; l Mean temperature; m Mean precipitation; n Layer; o Spatial effects

2.2.5 Model results

Considering the random effects component of the estimation model for incidence of Prostate cancer, the estimated standard deviation for spatial random effects is 0.378, indicating the presence of some spatial effects on the overall map. See Table S7 for details.

Before fitting the model, we pixelized the study region to construct prediction data. To ensure predictions for smaller areas, a grid was generated with a finer resolution, defined as 0.04 degrees latitude and longitude. This resulted in 599,225 grid cells, each measuring 4400m × 4400m, to capture the spatial resolution of the main covariates. The spatial distribution of the covariates were shown in Figure S3.

Table S7 Posterior estimates of hyperparameters and parameters of INLA-SPDE Bayesian spatial model of incidence of Prostate cancer in mainland China in 2016

Variable	Mean	Std	0.025quant	0.5quant	0.975quant	Mode
**Fixed effect**						
Intercept	1.814	0.027	1.761	1.814	1.867	1.184
Education	0.101	0.034	0.034	0.101	0.168	0.101
PerDI	0.262	0.030	0.204	0.262	0.319	0.262
SIpop	0.153	0.028	0.098	0.153	0.207	0.153
UrbanratePR	0.098	0.033	0.032	0.098	0.163	0.098
**Random effect**						
Range for w	0.532	3.663	0.014	0.102	3.744	0.023
Stdev for w	0.378	0.194	0.193	0.326	0.908	0.228
Precision for ID	4.089	0.312	3.532	4.068	4.759	4.013

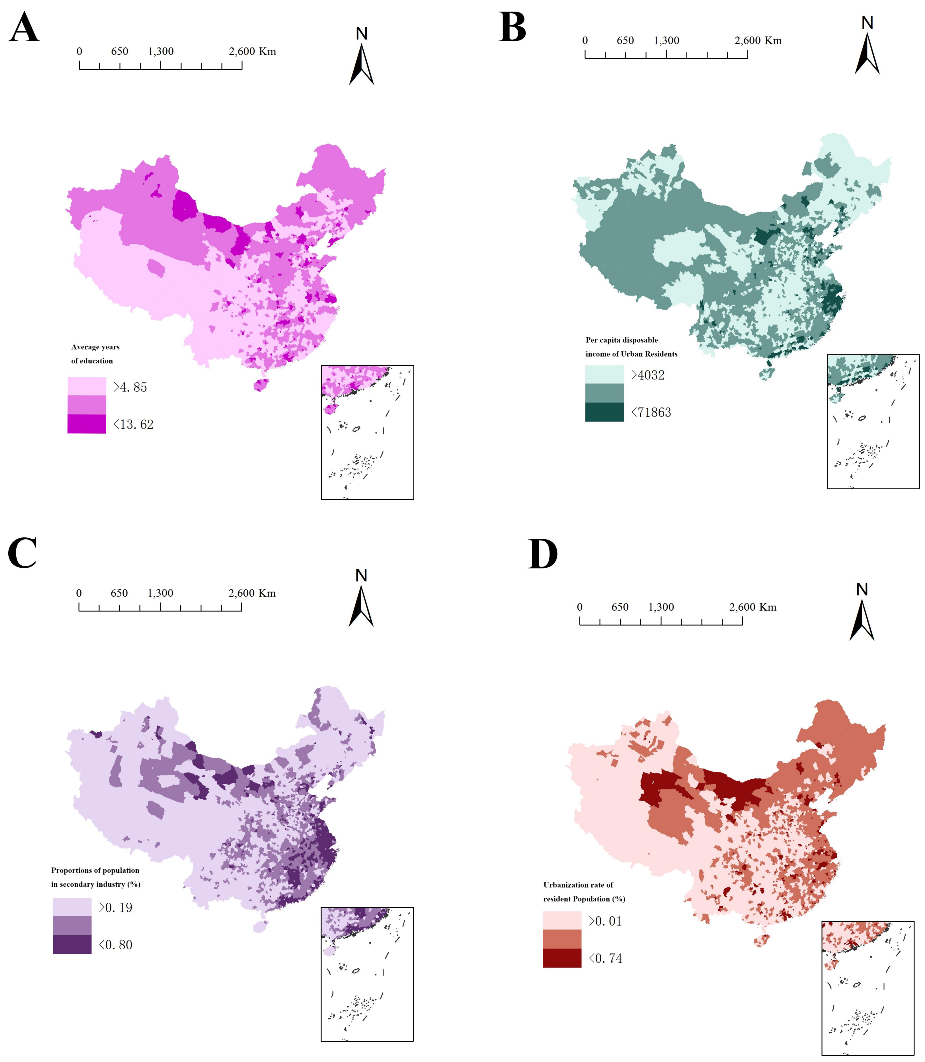

Fig. S3 Spatial Distribution of Covariates in Incidence Estimation Models (A Average years of education; B Per capita disposable income of Urban Residents; C Proportion of population in secondary industry (%); D Urbanization rate of Resident Population (%))

2.3 Model validation

We employed a 5-fold cross-validation method for model validation. All survey data were randomly divided into 5 subsets with similar sample sizes, with 4 subsets used as training data and the remaining one as validation data. The Bayesian geostatistical model was fitted based on the training data, and the estimated incidence rates were calculated at the locations of the validation data to assess the model's performance. We calculated the average of the results from the 5-fold cross-validation, with the root Mean Squared Error (RMSE) being 4.70, and achieving a 96.9%

accuracy in the 95% Bayesian confidence interval (BCI) for estimation model for incidence of Prostate cancer.

$$RMSE = \sqrt{\frac{\sum (es - ob)^2}{n}}$$

2.4 Sensitivity analysis

To assess the impact of model parameters on predictive performance, we performed a sensitivity analysis of Bayesian spatial models. We focused on the range parameter and sigma parameter settings and analyzed how variations in these parameters across various prior distributions affected the model's DIC, WAIC, and MLL.

We observed that the model for estimating male incidence of prostate cancer was particularly sensitive to changes in the sigma parameter, especially in regions of strong spatial correlation. When the sigma parameter was varied from 10 to 0.01, the model tended to overfit, which was reflected in the increase in DIC. Meanwhile, the range parameter affects to some extent the ability of the model to capture spatial structure. In the sensitivity analysis, we observed that the model showed better continuity at smaller spatial scales as the distance parameter decreased, which was confirmed by the decrease in DIC.

The results of the sensitivity analyses of the model for estimating incidence for Prostate cancer were shown in Figure S4. The figure showed the variation in model DIC for different combinations of range and sigma parameters. This visualization tool highlights the robustness of the model under specific parameter configurations.

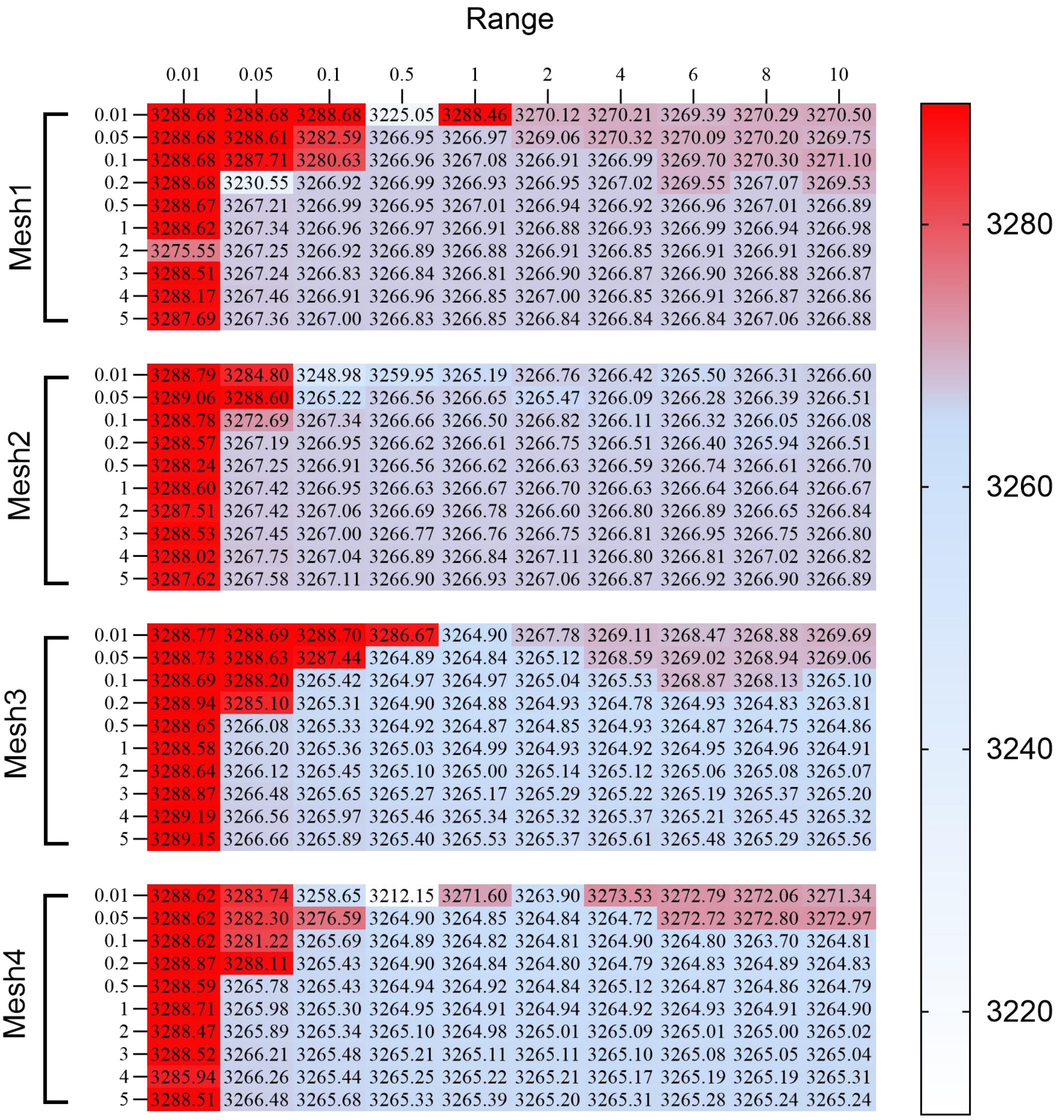

Fig. S4 Sensitivity Analysis Results for Range, Sigma, and Mesh Parameters in Prostate Cancer Incidence Models

2.5 Estimated incidence of Prostate cancer in resident population

The net migrant population was calculated as the difference between the resident population and the household- registered resident population, where a positive value indicated an inflow and a negative value an outflow. The number of Prostate cancer incident cases in the resident population was equal to the sum of the number of incident cases in the household- registered resident population and the net migrant population. The number of incident cases in the migrant population was calculated as follows:

$$N_{ij} = N_i \times W_{ij}$$

$$M_i = \sum_{j=1}^{31} \left(N_{ij} \times P_j\right)$$

N_{ij} denotes the number of migrant population moving from province j to province i among all migrant population in province i; N_i represents the total number of migrant population in province i; W_{ij} indicates the proportion of migrant population moving from province j to province i. The composition of migrant populations across provinces were calculated using data from the 2016 China Migrant Population Dynamic Survey, as shown in Figures S5. M_i refers to the number of incident/dead cases among migrant population in province i; P_j represents the incidence of the household-registered resident population in province j.

	Anhui	Beijing	Chongqing	Fujian	Gansu	Guangdong	Guangxi	Guizhou	Hainan	Hebei	Henan	Heilongjiang	Hubei	Hunan	Jilin	Jiangsu	Jiangxi	Liaoning	Inner Mongolia	Ningxia	Qinghai	Shandong	Shanxi	Shaanxi	Shanghai	Sichuan	Tianjin	Xizang	Xinjiang	Yunnan	Zhejiang	NA
Anhui	90.31	0	0.16	0.51	0.20	0.16	0.04	0	0	0.47	1.86	0.16	0.47	0.40	0.08	1.38	1.19	0.12	0	0	0	0.59	0.16	0.12	0.08	0.40	0.04	0	0.04	0.04	1.03	0
Beijing	6.32	0.11	0.78	1.21	1.64	0.80	0.20	0.23	0.03	23.52	13.64	5.46	4.25	2.21	2.61	2.93	1.87	3.59	3.48	0.20	0.03	10.94	4.91	2.15	0.14	3.36	1.35	0	0.32	0.26	1.44	0.03
Chongqing	0.44	0.20	67.43	1.04	0.04	0.96	0.36	1.65	0	0.40	0.80	0.16	1.81	1.93	0.08	0.48	0.76	0.12	0.36	0	0.16	0.84	0.48	0.32	0.08	17.07	0.08	0.16	0.28	0.64	0.84	0
Fujian	3.21	0.03	4.79	42.64	0.24	0.42	1.00	4.79	0.11	0.53	4.03	0.26	4.21	3.87	0.13	0.45	12.50	0.05	0.03	0	0	0.45	0.26	0.76	0	12.56	0.05	0	0	1.82	0.82	0
Gansu	2.53	0.05	0.83	1.10	67.34	0.28	0.09	0.14	0	1.06	4.96	0.32	2.99	1.47	0.32	1.52	1.01	0.37	0.37	0.37	0.60	1.38	0.51	3.08	0	3.54	0.05	0	0.51	0.23	3.03	0
Guangdong	1.77	0.02	2.55	2.49	0.28	29.55	13.58	3.03	0.40	0.20	4.60	0.34	7.25	14.97	0.30	0.44	6.51	0.18	0.12	0	0.04	0.54	0.20	0.86	0.04	7.73	0	0	0.08	1.29	0.60	0.02
Guangxi	1.66	0.04	0.68	2.46	0.04	3.33	72.88	0.95	0.38	0.42	0.87	0.30	1.48	6.85	0.19	0.68	2.19	0.26	0.15	0	0	0.68	0.11	0.23	0	1.29	0.04	0	0.04	0.23	1.51	0.08
Guizhou	1.16	0.04	5.75	1.90	0.07	0.41	0.78	58.89	0	0.41	1.76	0.15	2.99	9.11	0.15	0.75	2.32	0.15	0	0	0	0.86	0.11	0.26	0	8.96	0.07	0	0	0.82	2.13	0
Hainan	2.84	0.38	1.56	2.65	0.62	7.38	3.50	0.99	42.45	0.66	5.06	2.74	4.87	5.73	0.71	0.66	4.59	1.09	0.28	0.05	0.05	1.09	0.52	0.57	0.05	7.48	0.19	0	0.14	0.24	0.85	0
Hebei	2.89	0.29	0.68	0.89	1.21	0.18	0.07	0.43	0	56.55	8.31	6.06	2.71	0.46	1.28	0.78	0.75	1.75	2.39	0.07	0.07	3.75	1.68	1.93	0.04	2.28	0.64	0	0.04	0.32	1.46	0.04
Henan	1.85	0.08	0.91	1.55	0.08	0.34	0.15	0.08	0	0.95	82.60	0.34	1.85	0.83	0.08	0.57	0.79	0.23	0.08	0.04	0.30	1.44	1.02	0.83	0	1.13	0.04	0	0.08	0.04	1.74	0
Heilongjiang	2.64	0.14	0.14	0.19	0.10	0.14	0	0	0	0.62	0.77	80.86	1.15	0.14	5.08	0.34	0.29	1.63	2.16	0	0.05	2.06	0.10	0.14	0.10	0.43	0.10	0	0	0	0.62	0
Hubei	1.78	0.08	5.68	1.24	0.46	0.43	0.31	0.31	0	0.66	6.92	0.23	69.80	3.13	0.08	0.81	2.09	0.23	0.04	0.04	0.15	0.62	0.54	0.35	0.04	1.59	0.08	0	0.04	0.23	2.01	0.04
Hunan	0.66	0	0.16	1.52	0.08	0.51	0.39	0.43	0.08	0.23	1.01	0.08	2.18	87.95	0.04	0.31	1.98	0.08	0	0	0.04	0.19	0.04	0.23	0	0.86	0	0	0	0.04	0.93	0
Jilin	1.70	0.05	0.19	0.34	0.15	0.05	0	0	0	0.68	1.56	12.41	0.83	0.29	69.44	1.27	0.34	3.99	2.19	0	0.10	2.53	0.10	0.10	0	0.78	0.05	0	0.05	0	0.83	0
Jiangsu	27.39	0	1.12	1.49	0.72	0.19	0.14	1.28	0.07	0.47	8.31	0.72	2.71	1.59	0.42	33.63	3.20	0.44	0.14	0.05	0.19	3.92	0.35	1.77	0.86	5.14	0	0	0.07	1.14	2.43	0.02
Jiangxi	3.39	0.09	0.61	4.19	0.94	0.57	0.38	1.08	0	0.52	2.03	0.61	3.30	5.37	0.24	1.32	67.48	0.14	0.05	0.05	0.47	1.04	0.05	0.71	0.05	0.99	0.05	0	0	0.14	4.15	0
Liaoning	2.12	0	0.63	0.86	0.28	0.16	0.04	0.08	0.12	1.77	3.58	20.75	0.43	0.39	10.14	1.26	0.67	41.71	5.35	0.04	0	5.03	0.86	0.47	0.12	1.89	0.12	0.04	0.04	0.12	0.94	0
Inner	0.55	0.08	0.16	0.20	1.66	0	0	0.16	0	3.88	1.39	3.09	0.36	0.20	1.11	0.16	0.12	0.71	76.26	0.59	0	1.31	3.88	2.97	0	0.36	0.08	0	0	0.08	0.67	0
Ningxia	1.86	0.05	0.62	0.29	15.38	0.05	0.05	0.05	0	1.62	5.44	0.43	1.34	0.57	0.05	0.76	0.53	0.19	1.24	56.97	0.05	1.34	1.10	6.83	0	1.48	0	0	0.10	0.05	1.58	0
Qinghai	3.51	0.05	1.46	0.59	11.04	0.14	0.05	0	0.05	1.78	10.31	0.09	3.60	2.92	0.18	2.10	1.05	0.14	0.14	0.36	44.94	2.05	1.09	3.10	0	6.75	0	0.05	0.05	0.32	2.10	0
Shandong	1.12	0	0.10	0.48	0.17	0.17	0.03	0.07	0	1.53	1.67	3.61	0.54	0.17	1.29	0.48	0.54	0.27	0.54	0	0.03	84.81	0.24	0.31	0.03	0.31	0.03	0	0.07	0.03	1.33	0
Shanxi	2.99	0.15	0.81	1.07	0.50	0.15	0	0.12	0	5.79	7.67	0.35	1.23	0.38	0.27	1.65	1.07	0.31	3.80	0.04	0.19	1.46	64.90	1.96	0	0.84	0.23	0	0.08	0.19	1.80	0
Shaanxi	2.03	0.04	1.03	1.76	2.68	0.08	0.04	0	0.04	1.45	6.54	0.38	2.14	0.80	0.19	0.96	0.99	0.23	1.53	0.19	0.19	1.87	2.75	67.74	0	2.87	0	0	0.15	0.11	1.22	0
Shanghai	28.56	0.21	2.02	2.89	1.13	0.71	0.45	1.04	0.21	0.77	10.26	1.43	4.05	2.44	0.86	16.99	5.44	0.89	0.30	0.12	0.03	4.52	0.60	1.25	0	6.55	0.18	0	0.03	0.71	5.33	0.03
Sichuan	0.34	0.04	4.69	0.72	0.34	0.27	0.15	0.27	0.04	0.69	0.99	0.19	0.95	1.03	0.08	0.38	0.88	0.11	0.08	0.04	0.30	0.50	0.50	0.23	0.11	83.70	0.04	0.23	0.11	0.91	1.10	0
Tianjin	5.93	0.66	0.54	1.78	1.74	0.23	0	0.35	0	20.70	10.19	8.29	3.91	1.01	3.41	3.02	0.97	2.48	2.17	0.23	0.12	23.80	2.29	1.12	0.08	2.13	0.58	0	0.12	0.16	1.98	0
Xizang	0.65	0	3.81	0.39	7.05	0.35	0.22	0.74	0	0.74	4.41	0.22	1.12	0.65	0.26	0.48	0.26	0.13	0.04	0.30	1.25	0.78	0.52	1.64	0	49.91	0	22.49	0.26	0.87	0.48	0
Xinjiang	3.17	0.08	3.12	0.41	14.54	0.14	0.11	0.41	0.03	1.20	16.40	0.63	2.10	1.01	0.22	1.67	0.33	0.08	0.11	2.24	0.71	2.10	0.41	3.17	0.03	13.94	0.08	0	28.40	0.57	0.87	1.69
Yunnan	1.57	0.07	4.66	2.50	0.04	0.56	0.89	5.52	0.04	0.30	1.68	0.37	3.17	8.16	0	1.38	3.02	0.19	0.07	0	0.04	0.41	0.11	0.26	0.04	10.47	0	0	0.07	51.51	2.91	0
Zhejiang	19.83	0.06	3.41	1.86	0.56	0.23	0.88	10.59	0.04	0.43	9.60	0.36	5.27	5.38	0.23	2.69	11.63	0.24	0.11	0.02	0.15	1.71	0.17	1.07	0.08	7.36	0.02	0	0.02	4.03	11.95	0.02

Fig. S5 Composition of the Male Immigrant Population in 31 Provinces of Mainland China

Formula 1:

Net migrant population = Resident populations - Household-registered populations.

(1) The composition of the immigrant population by province were calculated using data from the 2016 China Migrants Dynamic Survey, as presented in Formulas 2 and Figure S5.

Formula 2:

$D_{jk_i} = \frac{N_{k_i}}{\sum_{j}^{31} N_j} \times 100$**,** in this formula, D_{k_i} represents the proportion of male population from province k_i among all male immigrant populations to province j. N_{k_i} denotes the number of male immigrant populations from province k_i while N_j signifies the total male immigrant populations in province j.

Formula 3:

$C_j = \sum_{i=1}^{31} \left(N_{k_i} \times P_{k_i}\right)$, in this formula, C_j represents the number of incident cases in the male migrant population of province j, N_{k_i} denotes the number of male migrant population from province k_i, while P_{k_i} indicates the incidence of the household-registered populations in province k_i.

Weights used to assess the magnitude of the impact of the migrant population on the incidence of prostate cancer in the resident populations.

Formula 4:

Weights = P_j/P_0 , in this formula, P_j represents the weighted prostate cancer incidence of the migrant population; P_0 represents the prostate cancer incidence of the household-registered population.

(2) The incidence of prostate cancer for resident populations was obtained by combining data from household-registered population and migrant population.

Formula 5:

The number of incident cases of prostate cancer among the resident populations = the number of incident cases among the registered resident population ± the number of incident cases among the migrant population.

(5) Age-standardized incidence rates of prostate cancer were indirectly calculated using Formula 5.

Formula6:

$$\text{Standardized incidence Rate (SIR)} = \frac{\sum \text{Observed Cases}}{\sum \text{Expected Cases}} \times \text{Reference Population Incidence Rate}$$

Appendix 3. Result

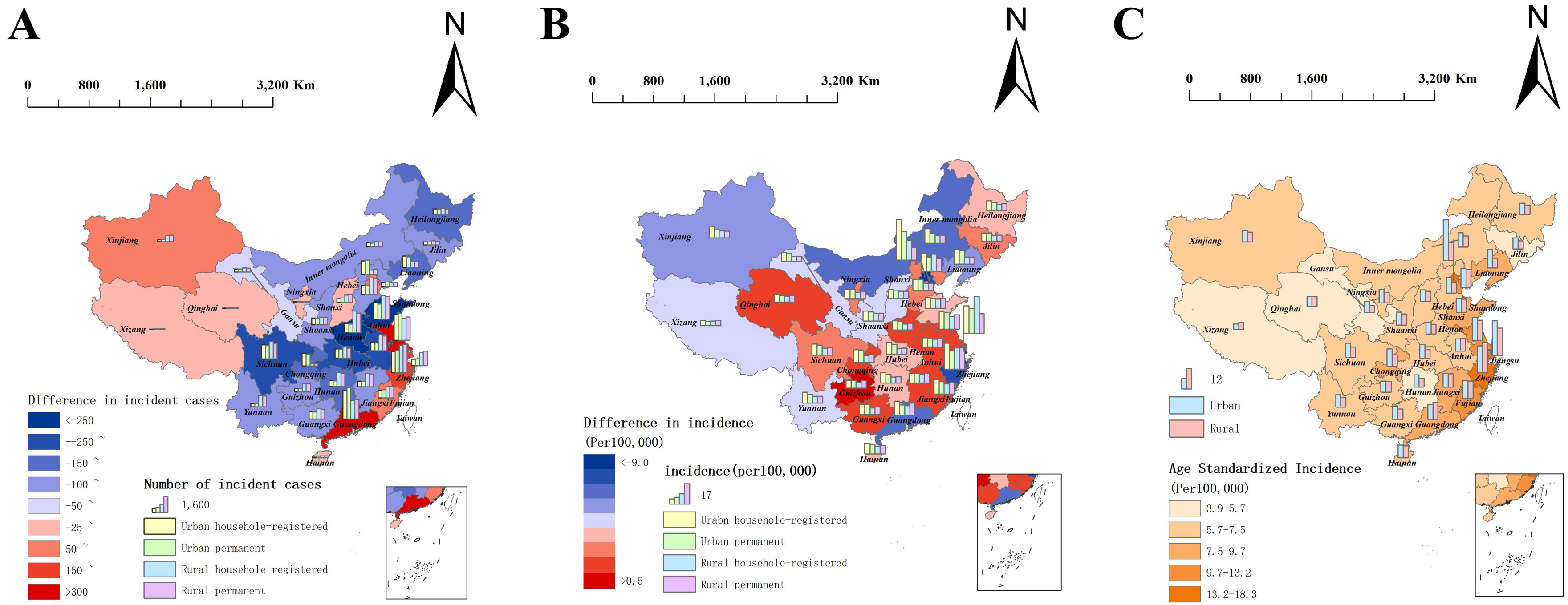

Fig. S6 A Difference in the Estimated Incident Numbers; B Difference in the Estimated Incidence Rates; C Standardized Incidence Rates and the Difference between RP and HRP

HRP household-registered population

RP resident population

Table S8 Spatial Clustering of Prostate Cancer Incidence: Local Autocorrelation Analysis

Cluster	Province	Number of Counties	County-level coverage（%）	population	Population coverage（%）
HH	Anhui	31	25.8	7,892,957	12.5
HH	Beijing	15	88.2	9,973,996	45.1
HH	Fujian	66	71.0	15,924,989	39.9
HH	Guangdong	34	23.4	11,657,149	10.1
HH	Hebei	30	16.9	6,503,739	8.9
HH	Hubei	5	4.3	2,673,600	4.4
HH	Jiangsu	73	67.6	30,754,648	38.4
HH	Jiangxi	13	11.7	3,057,423	6.5
HH	Liaoning	41	36.0	9,517,251	22.8
HH	Inner Mongolia	12	10.4	1,612,338	6.5
HH	Shandong	11	7.2	3,096,547	3.2
HH	Shanghai	16	94.1	12,515,117	50.0
HH	Sichuan	2	1.0	1,477,528	1.7
HH	Tianjin	15	88.2	7,849,132	48.9
HH	Zhejiang	90	89.1	30,927,356	50.0
HH	Chongqing	6	15.4	2,447,301	7.9
HH	Total	460	27.9	157,881,071	17.9
LL	Anhui	7	5.8	4,555,436	7.2
LL	Gansu	72	72.0	11,084,599	40.3
LL	Guangxi	54	43.2	8,794,171	17.2
LL	Guizhou	64	66.0	11,916,673	33.0
LL	Hebei	39	21.9	8,181,264	11.2
LL	Henan	119	68.0	39,225,088	41.0
LL	Heilongjiang	83	61.9	13,777,458	40.4
LL	Hubei	59	50.4	16,026,556	26.5
LL	Hunan	82	60.3	23,731,586	34.1
LL	Jilin	36	52.2	7,478,908	32.6
LL	Liaoning	1	0.9	296,911	0.7
LL	Inner Mongolia	24	20.9	3,888,593	15.7
LL	Ningxia	10	37.0	1,481,741	21.5
LL	Qinghai	1	1.9	2,107,564	34.8
LL	Shandong	14	9.2	5,612,917	5.9

LL	Shanxi	79	61.7	13,005,378	34.7
LL	Shaanxi	58	49.6	8,936,615	22.4
LL	Sichuan	62	30.4	7,857,920	9.3
LL	Xizang	69	85.2	2,041,340	48.8
LL	Xinjiang	35	28.7	5,233,982	18.0
LL	Yunnan	111	76.6	19,342,280	39.9
LL	Chongqing	9	23.1	2,404,781	7.8
LL	Total	1,088	42.7	216,981,762	22.1
